# Bone regeneration in surgically created defects filled with autogenous bone: an epifluorescence microscopy analysis in rats

**DOI:** 10.1590/S1678-77572010000400005

**Published:** 2010

**Authors:** Marcos Heidy GUSKUMA, Eduardo HOCHULI-VIEIRA, Flávia Priscila PEREIRA, Idelmo RANGEL-GARCIA JUNIOR, Roberta OKAMOTO, Tetuo OKAMOTO, Osvaldo MAGRO FILHO

**Affiliations:** 1 DDS, MSc, Discipline of Oral and Maxillofacial Surgery, Department of Surgery and Integrated Clinics, Araçatuba Dental School, São Paulo State University (UNESP) Araçatuba, SP, Brazil.; 2 DDS, MSc, PhD, Discipline of Oral and Maxillofacial Surgery, Department of Surgery and Integrated Clinics, Araçatuba Dental School, São Paulo State University (UNESP) Araçatuba, SP, Brazil.

**Keywords:** Fluorescent dyes, Bone transplantation, Bone regeneration

## Abstract

Although the search for the ideal bone substitute has been the focus of a large number of studies, autogenous bone is still the gold standard for the filling of defects caused by pathologies and traumas, and mainly, for alveolar ridge reconstruction, allowing the titanium implants installation.

**Objectives:**

The aim of this study was to evaluate the dynamics of autogenous bone graft incorporation process to surgically created defects in rat calvaria, using epifluorescence microscopy.

**Material and methods:**

Five adult male rats weighing 200-300 g were used. The animals received two 5-mm-diameter bone defects bilaterally in each parietal bone with a trephine bur under general anesthesia. Two groups of defects were formed: a control group (n=5), in which the defects were filled with blood clot, and a graft group (n=5), in which the defects were filled with autogenous bone block, removed from the contralateral defect. The fluorochromes calcein and alizarin were applied at the 7th and 30th postoperative days, respectively. The animals were killed at 35 days.

**Results:**

The mineralization process was more intense in the graft group (32.09%) and occurred mainly between 7 and 30 days, the period labeled by calcein (24.66%).

**Conclusions:**

The fluorochromes showed to be appropriate to label mineralization areas. The interfacial areas between fluorochrome labels are important sources of information about the bone regeneration dynamics.

## INTRODUCTION

The rehabilitation of the stomatognathic system is of great importance as it associated with the recovery of esthetic, functional and psychosocial recovery of the patients. Otherwise, the lack of bone in the alveolar ridge is a great challenge for the rehabilitation success. Although the search for the ideal bone substitute has been the focus of a large number of studies^5,9,24^, autogenous bone is still the gold standard^6,14^ for the filling of defects caused by pathologies and traumas^23^ and mainly, for the alveolar ridges reconstruction, allowing the titanium implants installation^2^. The osteoinduction and osteoconduction properties, biocompatibility, and impossibility of disease transmission are differential characteristics of the high success rates of autogenous bone^26,28^.

Studies on bone reconstruction aim at improving the quality response and increasing new bone formation, as well as accelerating this process^19^. The knowledge of bone regeneration dynamics in autogenous grafts and their cellular and molecular mechanisms collaborates to these purposes, and allows for the exchange between biology and clinics, thus reflecting in the success of rehabilitation.

Burchardt^4^ (1983) introduced the term "creeping substitution" to describe the process of autogenous bone graft incorporation. More recently, Pacifici, et al.^16^ (2002) proposed a simplified model of bone regeneration in autogenous grafts. Initially, the platelets present in the blood clot suffer a degranulation process and, within few hours, they release growth factors and transforming factors. These factors stimulate the onset of capillary regeneration, and bind to osteoblasts and stem cells, stimulating cellular mitosis and deposition of osteoid substance. The lack of blood supply elicits a cellular necroses response. After 3 days of graft implantation, it is possible to observe the presence of first capillaries colonizing the graft. Macrophages are attracted to the place where they proliferate and release more fibroblastic and endothelial growing factors. After 3 days, there is a progressive deposition of osteoid matrix. At 14 days, the local revascularization is completed and then the creeping substitution process starts, with the grafted bone being progressively replaced by newly formed bone. From the 4th to 6th week, mineralization occurs, resulting in an immature and disorganized bone. Finally, the immature bone (woven bone) will be gradually remodeled and resorbed, being replaced by a lamellar bone, according to the functional and masticatory stimulus.

Many methodologies have been used to observe the bone regeneration process^13,25^. The mineralization of the osteoid matrix constitutes an important step of this process, and the observation of this phenomenon by epifluorescence microscopy using fluorochromes may contribute to the evolution of bone regeneration dynamics concept.

Fluorochromes are fluorescent labels with calcium affinity, the most used being alizarin, calcein and oxytetracycline. When different types of fluorochromes are injected in the organism at different moments of ossification, they bind to the available calcium that is precipitating in the mineralization areas. With the aid of filters that catch specific wavelengths for each fluorochrome, it is possible to visualize the mineralized areas in different colors for each period.

Methodologies using fluorochromes have been more frequently used in bone biology researches^7,12^, and studies using fluorochromes to evaluate the dynamics of autogenous bone grafts incorporation are needed. Therefore, aim of this study was to evaluate the autogenous bone graft regeneration dynamics in surgically created defects in rat calvaria, using epifluorescence microscopy. The possible contributions of autogenous bone to bone defect regeneration were also investigated.

## MATERIAL AND METHODS

This study was conducted in accordance with the ethical Principles for Animal experimentation adopted by the Brazilian College of Animal experimentation (COBeA), have been approved by the Animal experimentation ethics Committee of the Veterinary Medicine Faculty of Araçatuba/UNeSP, Brazil.

**Figure 1:**
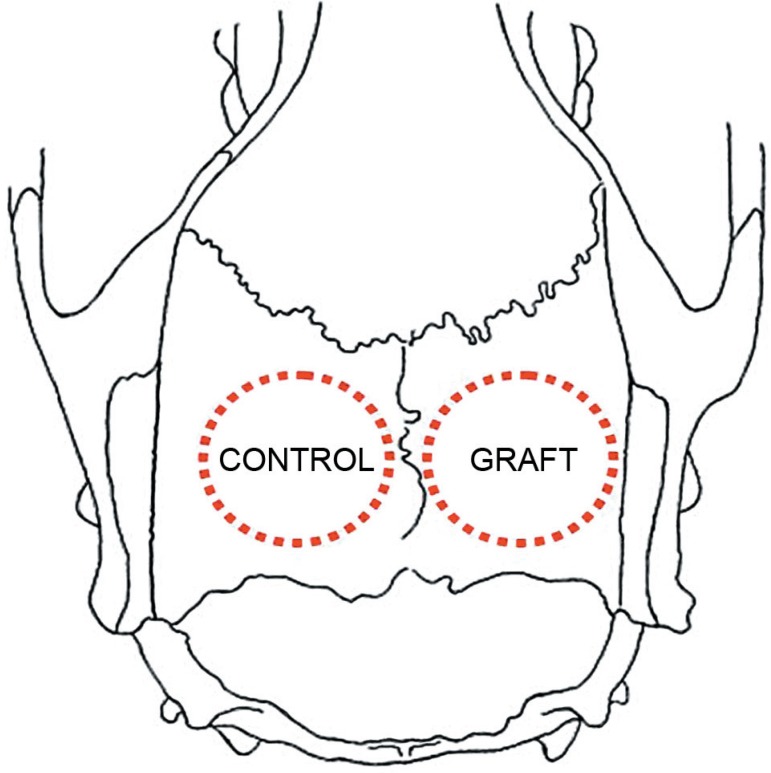
Rat calvaria scheme in an upper view. The 5-mmdiameter surgical bone defects were localized bilaterally at the parietal bones and were filled as illustrated

**Figure 2:**
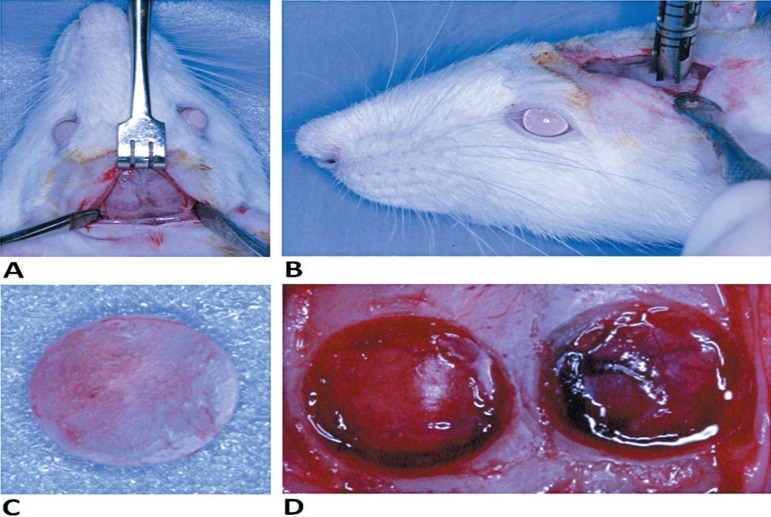
Surgical procedure. A) Surgical access. A linear incision in an anterior-posterior direction was made in the median region of calvaria and the dermoperiostic was detached. B) Trephine bur used to prepare the bone defects. C) The bone block removed from the left side was positioned in the right side defect. D) An approximate view of the defects to be filled

For the present study, 5 adult male Wistar rats (*Rattus novergicus albinus,*) aged 3 to 4 months and weighing 200 to 300 g were used. The animals received general anesthesia by the combination of ketamine hydrochloride (Dopaser -Laboratórios Calier S.A. Barcelona, Spain) and xylazine hydrochloride (Rompum; Bayer S.A. Saúde Animal, Porto Alegre, RS, Brazil) intramuscularly. A linear incision in an anterior-posterior direction was made in the median region of the calvaria and the dermoperiostic was detached. Two 5-mm-diameter bone defects were prepared with a trephine bur in each animal, being one in each parietal region. Two groups of defects were formed: a control group (n=5), in which the defects were filled with blood clot, and a graft group (n=5), in which the defects were filled with autogenous bone block, removed from the contralateral defect (Figures 1 and 2).

**Figure 3:**
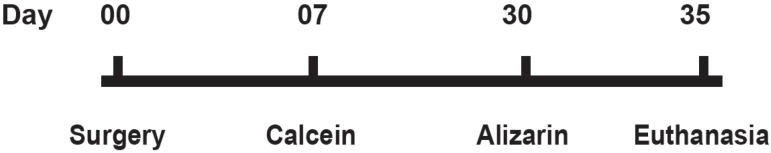
Schematic presentation of the application of calcein (7th postoperative day) and Alizarin (30th postoperative day) application (20 mg/kg body weight, IM)

The fluorochromes calcein and alizarin (Sigma Chemical, St. Louis, MO, USA) were applied intramuscularly at the 7th and 30th postoperative days, respectively, at a dose of 20 mg/kg body weight^17^, as presented schematically in Figure 3. The animals were killed by anesthetic overdose at the 35th postoperative day.

**Figure 4:**
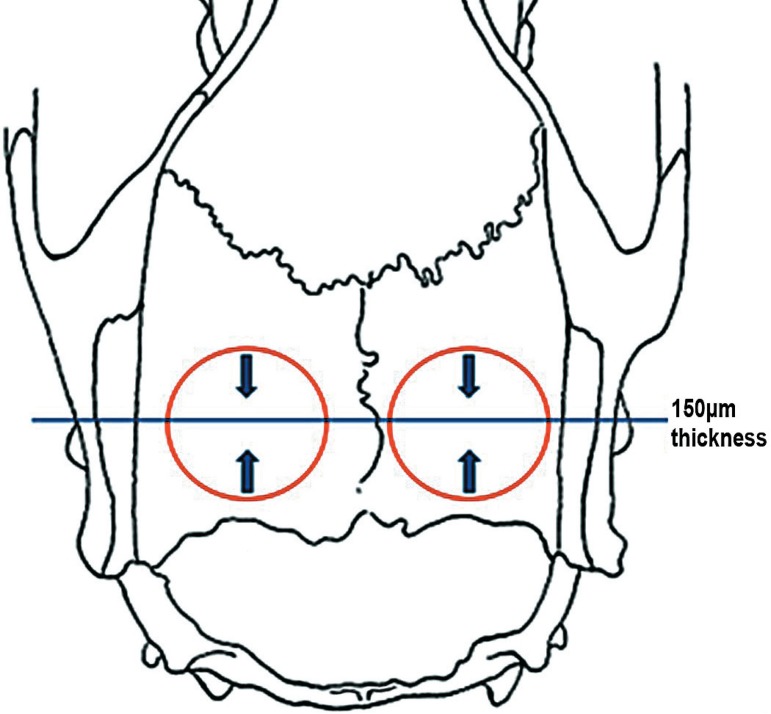
Preparation of the 150-μm-thick sections. The arrows indicate the wear direction to the center of the defect

The pieces were processed according to the protocol described by Maniatopoulos, et al.^11^ (1986). After inclusion in resin, blocks with the pieces were ground to a thickness of 150 µm (Figure 4).

**Figure 5:**
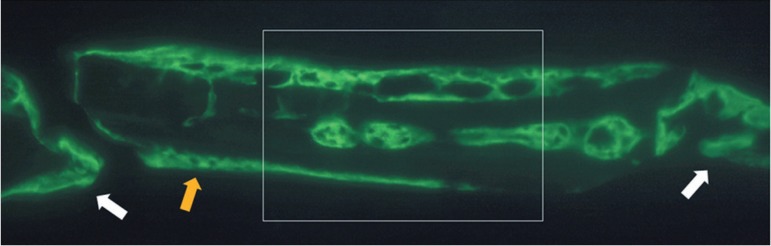
Standardization of the analyzed area. The square lines delimit the center of the defect, selected to be the analyzed area. The yellow arrow show the block graft and the white arrows show the boards of the defect

An epifluorescence microscope (Leica Aristoplan, Leica Microsystems, Wetzlar, Germany) with specific filters for each fluorochrome was used for evaluation of the results. Photomultiplicator filters of 488 nm wavelength for calcein and 594 nm wavelength for alizarin were used. For image acquisition, a digital camera was coupled to the epifluorescence microscope (Leica DFC 300 FX, Leica Microsystems, Wetzlar, Germany), whoch was connected to a computer (Pentium IV, Program IM 50 – Leica Microsystems, Wetzlar, Germany). For standardization of the analysis, only the central region of the defects was acquired (Figure 5). This criterion was established considering the difficulty to visualize the whole extension of defect with the alizarin filter in 4x increase and considering the low quality of image in this magnification.

**Figure 6:**
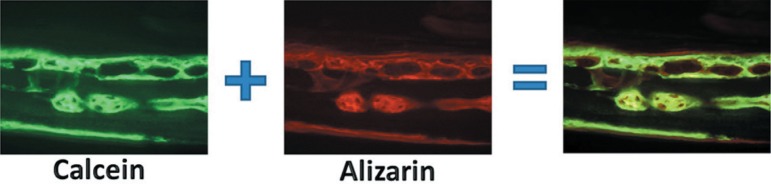
Image superposition scheme. The calcein and the alizarin images were superimposed in a computer program to obtain the final studied images

In each slide, two images were acquired, one labeled for calcein and one for alizarin. The images were saved in a digital file. The acquired images were superimposed using the Adobe Photoshop 7.0.1 (Adobe Systems Incorporated, San Jose, CA, USA) (Figure 6) to illustrate the dynamics of bone repair.

**Figure 7:**
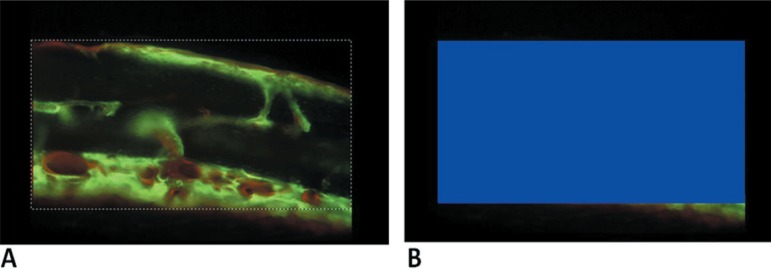
Total area (TA) standardization. A) The dashed lines delimit the area; B) Imagelab 2000® program calculated the area in pixels

**Figure 8:**
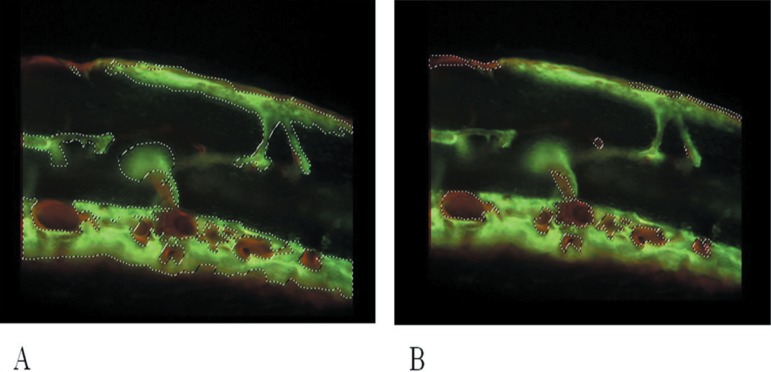
Calcein (A) and Alizarin (B) labeled areas delimited by dashed lines. (Imagelab 2000®)

In the quantitative analysis, Imagelab 2000 software (Canborough, Wellandport, Ontario, Canada) was used. Considering that the total area (TA) of the defect in the analyzed regions may correspond to the area occupied by calvaria bone in the same region before defect preparation, the TA was determined as follows: the defect that presented the greater heigh of labeling in the lateral side of the image was established as the pattern. From the most superior and the most inferior points of the labels in the lateral board that presented a greater height, two parallel lines were traced towards the other lateral board, forming a rectangle delimitating the TA (Figure 7a). The program calculation spreadsheet resource was used to obtain the TA value (Figure 7b), considering that the calvaria bone thickness was similar to all the animals included in the experiment. The calceinlabeled regions (green) (Figure 8a) and alizarinlabeled regions (red) (Figure 8b) were delimited and the areas were calculated in pixels. Only the most intensively labeled areas were considered (Figure 8). The TA was considered as 100% and the percentage of each labeled area was calculated for each group. Five percent values were obtained for each group (n=5) and averaged to obtain the final value of mineralized area in each analyzed period. The mean values were analyzed statistically by the t-test and Mann-Whitney test, using the Sigma Stat software, version 3.1. (Systat Inc., Chicago, IL, USA)

## RESULTS

**Figure 9:**
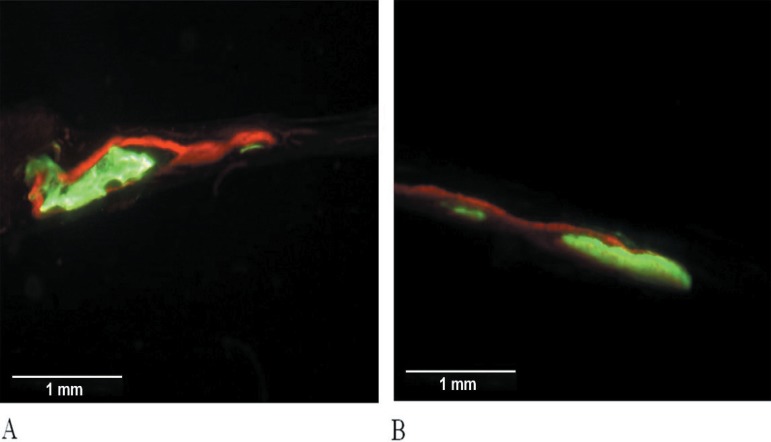
Fluorochrome-labeled areas in the Control Group. Three of five animals did not show labeled areas (green = calcein ; red = alizarin)

**Figure 10:**
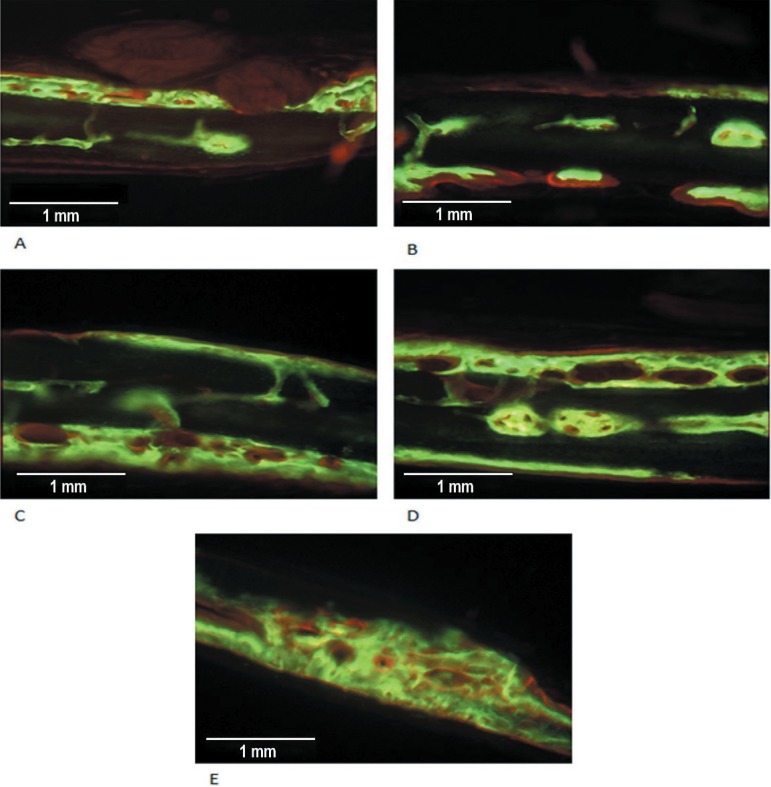
Fluorochrome-labeled areas in the Graft Group. The five animals showed strong labeled areas (green = calcein; red = alizarin)

The obtained images showed areas labeled in green and red that represent regions of calcium precipitation, labeled by fluorochromes in different moments of tissue mineralization. The calcein labeling (green) represents the regions where calcium precipitated from the 7th to 30th day. The alizarin labeling (red) represents the regions where calcium precipitated from the 30th to 35th days (Figures 9 and 10).

In the Control Group, the two animals presented images of isolated sites of mineralization in the center of the defect. In the other 3 animals, labeling by fluorochromes was not observed. The sites of mineralization presented similar characteristics with areas intensely labeled mainly by calcein, in contrast with areas of less intense labeling. Alizarin showed intense labeling, revealing a pattern of laminar formation, covering the calcein labels and located mainly between this and the periosteal surface (Figure 9).

In the Graft Group, all the slices of this group showed significantly more calcein and alizarin labeling than the Control Group. Otherwise, calcein (in green) presented labeling sites with greater density and a tendency of bone formation in blocks. Alizarin (in red) was characterized by less intense labeling with more laminar formation, primarily in the periosteal surfaces, covering the calcein labels (Figure 10). The images revealed that one of the surfaces (periosteal or dural) of the defects always showed more extensive and dense labels than the other, primarily by calcein. The alizarin labels in these surfaces were always located externally to those of calcein (Figure 10).

Large areas with no fluorochrome label or with weak labels (which were not considered) in the medullar region characterized this group (Figure 10), except for one slice, where the labels seems to be fused, forming a single block in the periosteal surface (Figure 10E). Otherwise, in these areas, some spread mineralization sites were observed (Figure 10).

The thickest areas labeled with calcein seemed to be excavated with cavitations apparently lined by alizarin labels. In some slices, projections seemed to be extending from the alizarin labels, joining the cavitations (Figure 10C and Figure 10D).

## DISCUSSION

Bone is a specialized mineralized connective tissue composed by 33% of organic matrix (primarily collagen type I) and 67% of inorganic matrix (hydroxyapatite crystals)^27^.

The incorporation of autogenous graft process involves the steps of induction, revascularization, resorption, osteoid matrix production, mineralization and remodeling^4^. Mineralization involves numerous complex events that are not well understood. After osteoid matrix deposition by osteoblasts, the collagen fibers of the matrix presents areas of cracks in their molecules, where the calcium ions are linked to proteoglycans. Under enzymatic action, phosphoproteins occupy the place of proteoglycans, initiating the precipitation of calcium phosphate complex. This way, the formation of hydroxyapatite starts and the hydroxyapatite crystals gradually occupies the areas of cracks, expanding between the fibers and completely mineralizing the tissue^10^.

The action mechanism of fluorochromes is not well understood. Images obtained in the Graft Group (Figure 10) suggest that these labels link to calcium while it stays available in the osteoid matrix, before the formation of hydroxyapatite crystals. Therefore, the fluorochromes would act only on the tissues undergoing mineralization, not labeling already mineralized tissues. The absence of labels over the graft surfaces reinforces this hypothesis.

According to this autogenous bone graft incorporation process described, we may consider the labeled areas as new bone formation areas, which are undergoing mineralization of the osteoid matrix. Otherwise, care must be taken when the mineralization areas are related to bone formation. Parfitt, et al.^18^ (1990) concluded that the bone formation index calculated from the labels of tetracycline fluorochrome is under estimated in 10% approximately. Some factors may contribute to this discrepancy: the decline of the osteoblast activity during the short period of life^18^ may weaken tetracycline fixation in the final of mineralization^7^; the areas of osteoid matrix that are not mineralized do not retain sufficient amount of tetracycline to achieve the threshold of detection in the epifluorescence microscopy technique^18^; and matrix synthesis and mineralization temporarily cease during bone remodeling^7,12^. Although there is no study testing the discrepancy between calcein and alizarin, it is suggested that these factors must be considered.

**Figure 11:**
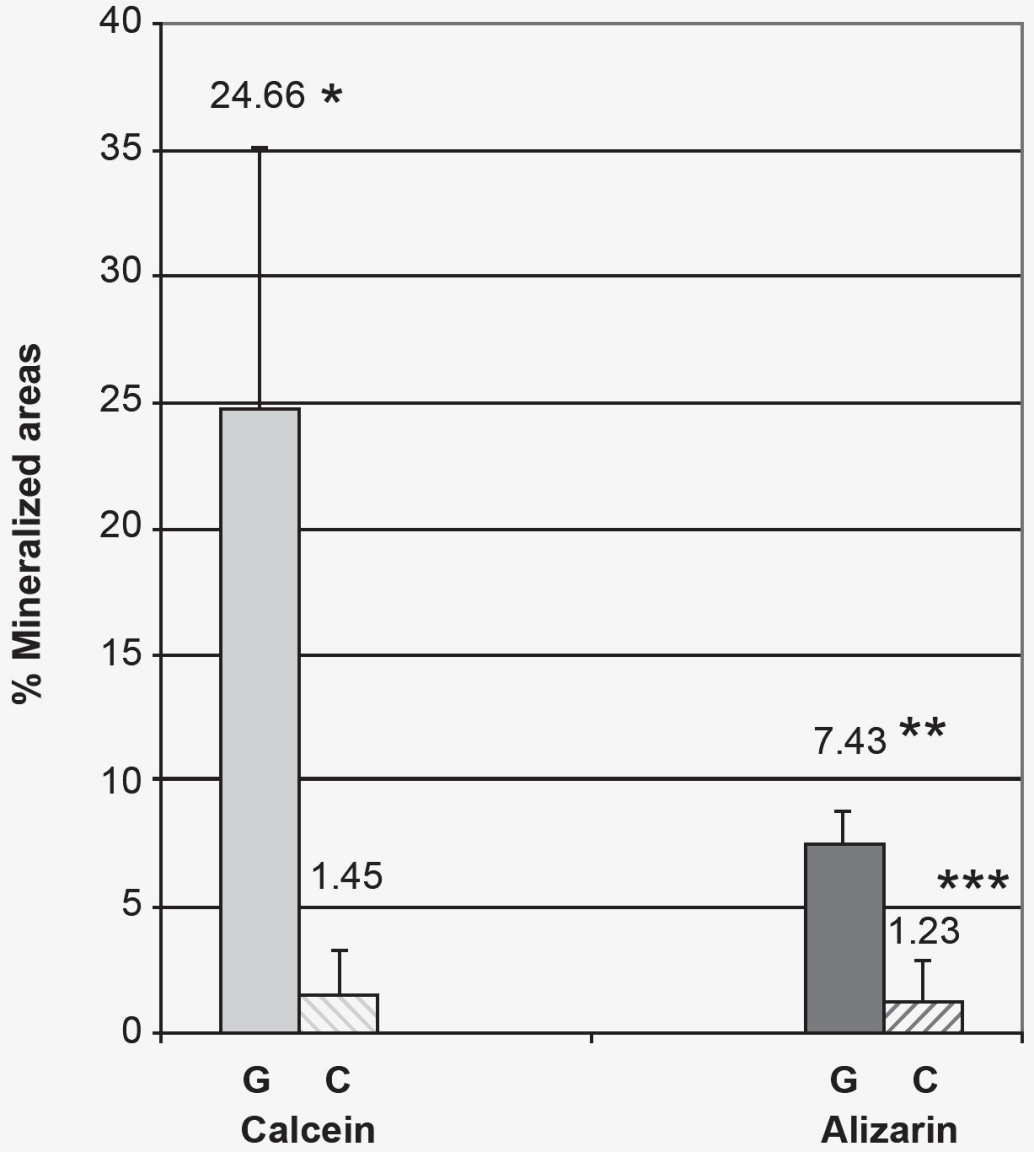
Percentage reached by calcein and alizarin in relation to the total area (TA). (G = Graft Group; C = Control Group). Results are expressed as mean ± standard deviation *Differs significantly from the Control group labeled by calcein (P = 0.008) (Mann-Whitney Test) **Differs significantly from the Graft Group labeled by calcein. (P = 0.008) (Mann-Whitney Test) *** Differs significantly from the Graft Group labeled by alizarin. (P = <0.001) (T Test)

**Figure 12:**
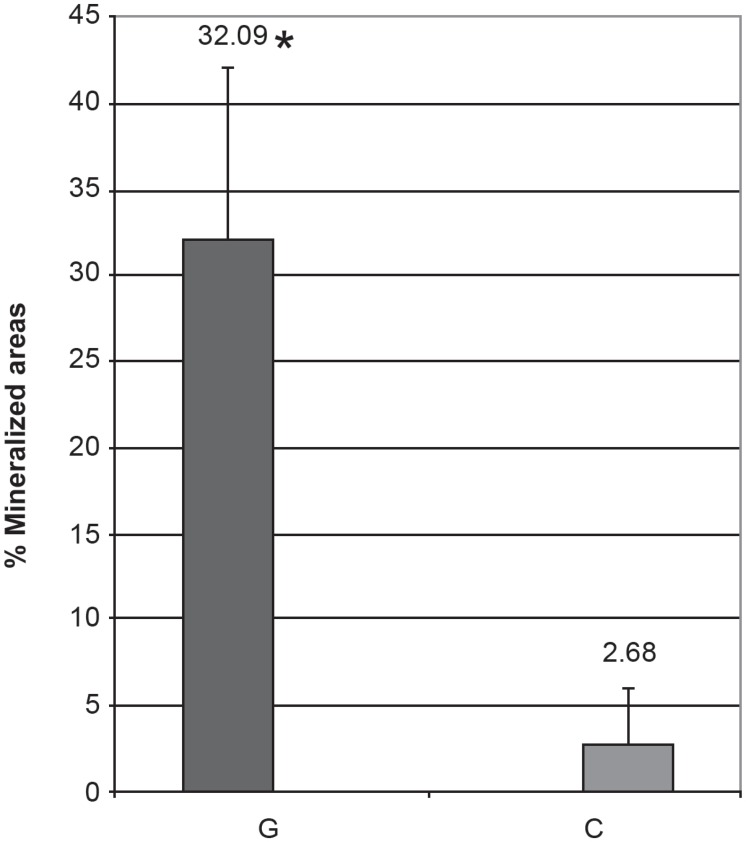
Sum of the areas labeled by calcein and alizarin in the Graft (G) and Control (C) groups. Results are expressed as mean ± standard deviation * Differs significantly from the Control Group. (P < 0.001) (T Test)

According to Frost^7^ (1983) and considering the periods of application of labels and sacrifice, the calcein labels must represent the areas that suffered mineralization between the 7^th^ and 30^th^ days, and the alizarin labels, the areas that mineralized between the 30^th^ and 35^th^ days, with the occurrence of areas of superpositioning between the two labels. The results of quantitative analysis seems, at least proportionally, in accordance to many published studies^20.22^. The quantity of labels in the Graft Group (32.09%) showed a regenerative potential of autogenous bone^4,28^, and the weaker labels in the Control Group (2.68%) suggest a critical size defect^1,3,8,21,29^ (Figures 11 and 12).

When the labels of two fluorochromes are compared, some factors must be considered. O’Brien, et al.^15^ (2002)classified the fluorochromes according to their level of affinity to calcium. Alizarin was considered as having the greatest affinity, followed by xylenol, blue calcein, calcein and oxytetracycline. The predominance of calcein labels (24.66%) on alizarin labels (7.43%) in the Graft Group possibly reflects the time of action for each label (calcein = 23 days, alizarin = 5 days). (Figures 11 and 12) If the mineralization occurred in the same intensity during the evaluated period, and considering the greater affinity of calcium to alizarin, we should have had a more intense label for this fluorochrome. As alizarin caused more discrete labels, we can suppose that the greatest mineralization rate occurs between the 7^th^ and the 30^th^ day, which is the period of calcein action. However, it is not possible to determine the moment when mineralization started and until which moment it occurred, since this process might have started before the 7^th^ day and would continue after the 35th day, if the rats had not been sacrificed,.

The areas with more intense bright in calcein labels are the regions with greater calcium precipitation. It is supposed that these are the areas where the mineralization started. The limits regions where the calcein labels touch the alizarin labels, represents the region where the mineralization occurred in the 30^th^ day after the graft implantation. After these considerations, it is possible to clearly see in some images, nuclei of centrifugal mineralization in many sites of the graft (Figure 10A, 10B and 10D).

The features observed in the images and the more intense and dense label in one of the surfaces of the defect (periosteal or dural) might be due to the position that the bone block was placed in the defect, with the external cortical in direction to the periosteal or dural surface. As no standardization of this sense was made at the moment of surgery, and also by the difficulty imposed by the size of the obtained blocks, we believe that the surface with greater label corresponds to the external cortical of the bone block (Figure 10). The images indicate that the mineralization initiates by the surfaces (periosteal or dural) of the grafts and by sites of mineralization located in the medullar regions. These nuclei suggest an osteoinduction activity of the autogenous graft.

The regions of alizarin labels seems to be lining the cavities formed by the calcein labels and sending projections linking the cavitations (Figures 10C and 10D), possibly representing the formation of Havers and Volkmann channels, that shelter blood capillaries. These structures that had their formation initiated in the calcein period of action (between days 7 and 30) seem to become more mature after the 30th day.

## CONCLUSION

The fluorochromes used in the present study appeared adequate to label mineralization areas, and the interfacial areas between the labels of two fluorochromes revealed important information about the dynamics of bone regeneration in regions grafted with autogenous transplants. However, the obtained results cannot be extrapolated to the clinical conditions, considering the differences in evolution, metabolism and dimensions between man and rat. More studies are needed using more than two types of fluorochromes with experimental models of metabolism more similar to that of humans, and with a longer postoperative evaluation period.
